# Context-specific roles for IL-17 in tuberculosis

**DOI:** 10.1371/journal.ppat.1014054

**Published:** 2026-04-21

**Authors:** Mahlatse Maseeme, Liku B. Tezera, Mahdad Noursadeghi, Alasdair Leslie, Gabriele Pollara

**Affiliations:** 1 Africa Health Research Institute, Durban, South Africa; 2 University of KwaZulu Natal, College of Health Sciences, Durban, South Africa; 3 School of Clinical and Experimental Science, University of Southampton, Southampton, United Kingdom; 4 Institute of Infection, Immunity & Transplantation, University College London, London, United Kingdom; University of Queensland, AUSTRALIA

## Abstract

The number 17 is considered unlucky by some Italians as its Roman numerals – XVII – can be rearranged as *Vixi*, a Latin expression that can be interpreted as “my life is over”. In other contexts, the number 17 is associated with wisdom and success. This paradox holds true when it comes to the role of interleukin 17 (IL-17) in tuberculosis (TB). On one hand, immune correlates of protection studies have consistently identified IL-17 responses as key players in natural and vaccine induced protection against infection and disease. On the other, IL-17 has been proposed as a main driver of the immunopathology that underlies TB morbidity and mortality. Thus, while some researchers seek to develop novel TB vaccine approaches that promote IL-17 responses, others hunt for host-directed therapeutic approaches that block its activity. In this article, we attempt to address this conundrum and synthesise the main arguments supporting a role for IL-17 in the protection and pathogenesis of this deadly human infectious disease. Ultimately, as with superstition, whether the 17th interleukin is friend or foe in human TB is likely to depend on the context.

## Introduction

The bacillus *Mycobacterium tuberculosis* (Mtb) is the human pathogen that causes tuberculosis (TB) infection and disease. Active, symptomatic, TB disease still accounts for ≈10 million cases and almost two million deaths annually, making it the most common infectious cause of death worldwide (World Health Organisation 2024). TB disease predominantly affects the lungs and initiates a robust host immune response involving multiple cellular components and cytokines, characterised by a pathognomonic histopathological feature, the TB granuloma, an aggregate of cells surrounding Mtb bacilli, necrotic tissue or calcification [1].

The clinical outcome of Mtb infection is likely dependent on the fate of host-pathogen interactions within individual granuloma [2], with both the lack of bacteriologic containment and immunopathology contributing to the manifestation of clinical symptoms. Determining which immune components play protective or deleterious roles in the natural history of TB disease is critical to establish desirable properties of efficacious TB vaccines and targets for adjuvant host-directed therapies in TB. For TNFα, genetic deletion and pharmacologic inhibition have revealed a key role in preventing TB disease [3,4]. However, unmasking of TB disease by checkpoint inhibitor therapy [5] has also exemplified that exaggerated cytokine activity, such as through IL-6 signalling, can also contribute to TB disease manifestation [6].

Several lines of evidence now point to the cytokine IL-17 playing a pivotal role in TB, and in this review, we will focus on summarising the key aspects of IL-17 cell biology that relate to Mtb infection, integrating *in vitro* and *in vivo* work from both human and non-human studies that highlight the beneficial and detrimental roles for IL-17 cytokine activity in Mtb infection. By offering polarised views, we aim to challenge readers’ pre-conceptions in either direction, before concluding with an overarching context-specific model for the role IL-17 cytokines play in TB.

### The IL-17 cytokine family

It has been almost three decades since the proinflammatory IL-17 cytokine family was identified as an important player in both health and disease [7]. Although often referred to in the singular, there are 6 currently known isoforms of IL-17, giving rise to the homo- and heterodimeric cytokine family IL-17A to F. These IL-17 family members in turn signal via five IL-17 heterodimeric receptors, termed IL-17RA to E, which are composed of the widely expressed IL-17RA and another receptor, depending on the cell type. The signalling mechanisms employed by the IL-17 cytokine family have been extensively reviewed elsewhere [7].

Conventional αβ CD4 T cells differentiated to secrete IL-17A/F (Th17 cells) are a significant source of IL-17 in vivo, and the cytokines IL-1β, IL-6, IL-23 and TGF-β play a role in this process [7]. However, IL-17 cytokines also originate from CD8+  T cells, γδ T cells, NK cells, iNK cells, innate lymphoid cells (ILC), neutrophils, and mast cells [7–10]. Most studies investigating the role of IL-17 cytokines focus on IL-17A and IL-17F due to the high level of homology and the proximity of their gene loci in humans and mice. IL-17A & F bind the same receptor complex (IL-17RA and IL-17RC), which translates into highly overlapping biological function, including promoting the expression of antimicrobial peptides, upregulating production of neutrophil chemokines, and inducing the expression of tissue remodelling enzymes, such as matrix metalloproteinases (MMPs) [7]. Therefore, in this review, we will refer to both IL-17A and IL-17F as IL-17, unless specified.

## A protective role for IL-17 in TB

IL-17 cytokine activity has shown protective roles in the context of non-TB infections, including *Candida* infection in which both mice and in humans with a deficiency in IL-17 cytokine activity, either through genetic deletions or pharmacological inhibition, who consistently show severe susceptibility to mucocutaneous candidiasis [11]. The mechanisms underlying this protection include the recruitment and bactericidal enhancement of neutrophils [12], as well as the induction of antimicrobial peptides [13,14].

### Mouse studies

Mouse models of TB infection support a critical role played by IL-17 in controlling Mtb infection and preventing disease. Using an outbred, genetically diverse population of mice, low bacterial burden was associated with Th17 signatures, and it was notable that Th1 responses were low in the mice with Th17-associated protection [15]. A BCG vaccination study using similar models also found that mice protected from Mtb challenge following BCG vaccination had enhanced IL-17 responses, although in this case with IFNy co-expression [16]. Conversely, mice lacking Mtb-specific Th17 responses due to absence of the p19 subunit of the Th17-inducing IL-23 cytokine, were highly susceptible to TB disease at high inoculum doses after infection with the lab-adapted H37Rv Mtb strain [17], associated with a delay in neutrophil infiltration [18]. Separately, IL-17 responses were essential for protection from the virulent Mtb strain HN878 in mouse models, by signalling via the IL-17 receptor on non-haematopoietic cells to promote CXCL13 activity and T cell aggregation [19]. These findings in mouse models mirror Mtb strain-dependent differences in IL-17 cytokine production by human PBMC stimulated in vitro [20].

Further support for the importance of IL-17 activity stems from evidence that Mtb may have evolved a mechanism to limit Th17 development. Bone marrow-derived mouse dendritic cells infected with Mtb were observed to down-regulate the co-stimulatory molecule CD40, preventing Th17 polarisation in vivo [21]. Restoration of CD40 signalling on DCs enhanced Mtb-specific Th17 generation and reduced pathogen burden. Importantly, the Mtb-derived protease Hip1 was found to attenuate expression of the Th17-associated Notch ligand, Delta-like ligand 4 (DLL4) [22], suggesting that suppressing Th17 polarisation confers a survival benefit to the pathogen.

A protective role for IL-17 responses early in mouse Mtb infection is further strengthened by the pivotal role in vaccine protection played by this cytokine [23]. Greater protection offered by subcutaneous co-administration of BCG with a liposomal adjuvant was associated with the expansion of Mtb-specific lung Th17 cells and sustained IL-17 responses [24]. Similarly, subcutaneous vaccination with Mtb ESAT-6 peptide formulated with mycobacterial lipid adjuvant induced markedly superior Th17 responses in the lung and greater Mtb control, but these mice also experienced greater weight loss, indicating the careful balance between IL-17 immunity and pathology (addressed later in the review) [25]. Mucosal vaccination has gained significant traction, with the goal of promoting tissue memory responses. In the case of TB, intranasal BCG vaccination was superior to subcutaneous routes in protecting mice from subsequent Mtb challenge in an IL-17A- and neutrophil-dependent but IFNg-independent manner [26]. Similarly, protection against TB disease following mucosal vaccination adjuvanted by type II heat-labile enterotoxin, STING-activating cyclic dinucleotides or delta inulin microparticles was also found to be dependent on IL-17 [27–29]. Overall, the consistent association between protection and induction of IL-17 responses upon Mtb challenge strongly supports a protective role for early IL-17 responses following in vivo Mtb infection, at least in mouse models of TB.

### Non-human primate studies

A limitation of the evidence from mouse studies is that Mtb is not a natural pathogen of mice, and therefore this *in vivo* model may not faithfully recapitulate the pathophysiological spectrum of human Mtb infection. In contrast, the non-human primate model of Mtb infection more closely reflects the natural history of Mtb infection, including the immunological components of the TB granuloma and the development of lung cavities [2]. Moreover, paucibacillary Mtb infection of macaques can result in asymptomatic infection, commonly observed in humans [30]. This is associated with greater expansion of IL-17 and IFNγ co-expressing Th17 cells (also termed Th17.1 cells) predominantly in the lungs compared to blood [31], a response proportional to the inoculum dose [32]. Investigators have also leveraged the autonomy of individual TB granuloma responses in NHP, relating elevated IL-17 responses (especially Th17.1) in granulomas with better bacteriologic control (lower Mtb burden) [33]. These findings have been recapitulated with greater resolution using single-cell RNA-seq approaches, again demonstrating that a greater frequency of Th17.1-like cells is associated with lower granuloma-associated bacterial load [34]. More recently, the same group has narrowed these observations down to Mtb-specific T-cells using MHC class II tetramers, observing Mtb-specific T-cells enriched in NHP granuloma, with frequency of these cells expressing the Th1 and Th17 transcription factors T-bet and RORγT inversely correlating with bacterial burden [35].

Like the findings in mice, vaccine studies in the NHP model of TB support a protective role for IL-17. Standard intradermal injection of BCG provides some level of protection in NHPs although it does not appear to induce sterilising immunity. Nevertheless, NHPs with relatively good disease control were found to have elevated Th17 and Th1 responses in restimulated PBMC compared to non-controllers [36]. More recently, intravenous administration of BCG (ivBCG) has emerged as an exciting vaccine model of protective immunity in NHPs, generating sterilising immunity in some vaccinated animals. Like the above study, restimulated airway cells from NHPs that received a protective (high) ivBCG dose displayed elevated Th1 and Th17 signalling [37]. In dose-ranging ivBCG experiments, in which not all animals are protected, airway CD4 T-cells producing IL-17 were identified as a key correlate of immune protection [38]. Importantly, these responses were much less evident in blood, highlighting the importance of studying the site of disease where possible. Another alternative vaccination route for BCG, via the airways, has also been explored by several groups. Although not always successful [39], sterilising immunity to repeated low-dose pulmonary Mtb challenge has been achieved via mucosal BCG vaccination [40]. In this case, protection was again associated with an expansion of polyfunctional Th17 cells in the lung. Together with the mouse studies highlighted, NHP data are consistent with a potentially central role for a Th17-like response in both natural infection and vaccine-induced immunity under controlled experimental settings.

### Human studies

IL-17 production is a feature of human recall responses following Mtb infection, and it can be detected following ex vivo stimulation, in circulating aβ T cells, invariant T cells, MAIT cells, and γδ T cells [10, 41,42]. However, causal evidence supporting its protective role in human Mtb infection is limited. Several studies have shown lower circulating frequency of IL-17-producing T cell subsets in active TB compared to healthy controls [42,43], and greater IL-17 production following mitogen-stimulation of blood seen following culture conversion on TB antibiotic treatment [44]. Likewise, isoniazid preventative therapy of TB-exposed individuals (IGRA positive) restores Mtb-specific IL-17 responses [45]. More recently, a multimodal strategy including single-cell RNA-seq and flow cytometry demonstrated depletion of Th17-like cells in individuals prior to development of TB disease [46]. A similar Th17 signature was found to be significantly depleted in published bulk blood RNAseq data from Mtb-exposed adolescents in South Africa 1 year prior to the development of active TB disease [47]. Conversely, Mtb-specific, CD4+ T cell Th17 responses were found to be a feature of “resister” individuals that do not develop TB infection/disease despite prolonged exposure, although whether this IL-17 activity directly prevented the development of TB disease and through which mechanism remains unclear [48].

Human data on IL-17 responses from the site of TB disease is harder to obtain, but a greater frequency of IL-17-producing T cells was observed in pleural TB from mononuclear cells in the pleural space compared to blood [49], implying enrichment of these cells at the site of disease. More recently, lung explants from TB patients requiring surgery demonstrated evidence of lung tissue-resident CD4 T cells secreting IL-17, whereas the frequency of IL-17 production in the peripheral blood of these same patients was significantly lower [50]. In addition, the frequency of Mtb-specific IL-17-producing T-cells was inversely correlated with severity of TB disease [50]. In the same study, addition of exogenous IL-17 to a human granuloma model was associated with elevated levels of nitric oxide and attenuation of Mtb growth [50], suggesting a potential mechanism for promoting microbiological control in humans.

Finally, there is evidence from natural genetic variation that IL-17 activity plays a protective role in TB. Several case-control studies have identified associations between single-nucleotide polymorphisms (SNPs) in IL-17A and IL-17F gene promoters and the risk of developing TB disease [51–55]. Specifically, for the IL17A gene SNP rs2275913, the minor A allele results in greater IL-17 production by stimulated PBMC and is found with lower frequency in patients with TB disease compared to controls [55,56]. This observation has been reproduced in the Chinese Han population using an alternative IL17A SNP (rs8193036) that also affects IL-17 production and TB disease susceptibility [54]. These genetic associations support the notion that overall IL-17 cytokines form an important cornerstone of the host response to Mtb infection contributing to control of early or paucibacillary infection, and thus the prevention of disease.

## A detrimental role for IL-17 in TB

Despite the arguments supporting a protective role for IL-17 responses in Mtb infection, this section will put forward a counter view, centred on the absence of disease risk in individuals with attenuated IL-17 signalling activity, as well as the potential for IL-17 responses and its downstream effectors to drive significant tissue pathology, promoting lung damage and disease transmission [57]. Indeed, IL-17-mediated pathology has been proposed as a significant contributor to disease in mouse models of the chronic fungal infection paracoccidiomycosis, a condition also associated with granulomatous lung inflammation [58].

### Lack of protection offered by IL-17 in TB

Inborn errors of immunity have increasingly demonstrated the dependence on specific immunological components for protection against certain infectious diseases [59]. Complete autosomal recessive deficiency of IL-17 receptor subunits, IL-17RA or IL-17RC, which ablates IL-17 cytokine signalling, is associated with an increased risk of mucocutaneous candidiasis and staphylococcal, but not mycobacterial, infections [60,61]. A similar clinical phenotype has been described for individuals with genetic deficiencies in IL-17F cytokine [62] and mutations in the IL-17 receptor adaptor molecule ACT1 [63]. Gain-of-function mutations in STAT1 and bi-allelic loss-of-function mutations in RORC, the gene encoding the transcriptional factor for the Th17 lineage RORγT, both impair the differentiation of Th17 cells and are associated with increased risk of mycobacterial infections, but these defects also inhibit the activity of IFNγ [64], a cytokine indispensable for protection to mycobacterial diseases [65]. Genetic defects in the signalling of cytokine receptors that promote Th17 differentiation, IL-1 and IL-6, are associated with increased risk of pyogenic, but not mycobacterial, infections [6,59,66]. Comparable to the effect of inborn deficiencies in these pathways, autoantibodies to cytokines have recently been identified as playing a potentially significant causative role in the susceptibility of infectious diseases, illustrated by the association between IFNγ-targeting autoantibodies and increased risk of mycobacteria disease, including TB [67]. However, individuals with autoantibodies to IL-17 or IL-6, while susceptible to some infections, including mucocutaneous candidiasis for IL-17, are not susceptible to mycobacterial diseases [67].

The associations between IL-17 SNPs and protection from TB disease described earlier need to be interpreted with caution due to uncertainty around generalisability in all populations, as well as the lack of functional correlation between most SNPs and cytokine production. Moreover, the ‘protective’ rs2275913 SNP A allele that results in greater IL-17 production [68] is also associated with radiologically more severe TB disease [56], hyperinflammatory responses in other mycobacterial infections, such as BCG-induced osteitis [69], leprosy type 1 reactions [70], giant cell arteritis [71], ulcerative colitis [72] and chronic periodontitis [73]. Similarly, genetic variants that increase activity of the IL-1 & IL-6 pathways have also been associated with more severe TB disease [66,74]. More recently, a meta-analysis of 17 genome-wide association studies (GWAS) studies used Mendelian randomisation to attribute lower risk of TB disease with the IL-6 receptor allele that attenuates in vivo IL-6 signalling and closely phenocopies the effect of pharmacologic IL-6 antagonism [6]. This further supports the notion that elevated IL-6 activity may contribute to TB disease risk, although a functional relationship between this phenotype and greater IL-17 activity has not been established.

Biologic agents that inhibit the activity of IL-17 and cytokine regulators of Th17 differentiation have been used extensively to treat chronic inflammatory conditions, such as psoriasis and ankylosing spondylitis. However, despite extensive and ongoing post-licensing surveillance, an increased risk of TB disease has not been observed with secukinumab (targeting IL-17A) as well as biologics targeting Th17-differentiating cytokines [75], unlike TNF-blocking biologics [3]. Moreover, an *in vitro* model of Mtb dormancy showed reactivation using anti-TNF therapy (adalimumab), but not secukinumab [76], and IL-17 blockade in Mtb-infected mice does not result in elevated bacterial growth, unlike the effects of TNF inhibition [77].

### IL-17 as a driver of pathology in TB disease

Once TB disease is established, exaggerated activity of the IL-17 cytokine axis, including drivers of Th17 differentiation, may promote excessive inflammation and tissue pathology.

Several human chronic diseases are characterised by elevated IL-17 activity at the site of disease, including psoriasis and inflammatory bowel disease, and the central role played by this cytokine in driving pathological inflammation is illustrated by robust and sustained clinical responses achieved by biologics blocking the IL-17 cytokine axis [78]. Moreover, Th17 cells in TB lesions make up a greater proportion of the total T cell population than that observed in other diseases [79], and crucially this appears to translate into functional IL-17 activity within and beyond the macroscopic edge of human TB granuloma [80]. Nevertheless, these cross-sectional assessments cannot negate confounding from differences in disease duration and antigenic burden, an aspect overcome in studies using an antigen challenge model to interrogate in vivo human immune recall responses in TB patients. Repurposing the tuberculin skin test (TST) from use as a diagnostic investigation into a research tool [79,81,82] revealed that standardised in vivo antigen stimulation induced greater IL-17 responses at the site of TSTs in TB patients compared to healthy individuals with past Mtb exposure or cured TB [79]. Notably, this difference was not observed for other inflammatory cytokines such as IFNγ or TNFα [79]. The elevated IL-17 activity was accompanied by greater IL-1 and IL-6 cytokine responses at the site of TST, as well as from ex vivo-stimulated monocytes in active TB [79], indicating that monocytic infiltration to the site of host-pathogen interactions may generate a cytokine environment that favours in situ Th17 differentiation and IL-17 cytokine activity [79,83–86].

Parallels between mouse models and human TB allude to common mechanisms of immunopathology. Robert Koch’s attempt to treat TB disease with inoculation of mycobacterial extracts was not only clinically ineffective but also resulted in harmful exaggerated inflammation both at the site of inoculation and established disease [87]. Similarly, repeated mycobacterial antigen challenge through BCG vaccination of mice with established Mtb infection amplified inflammatory responses to the detriment of the host through exaggerated IL-17 and neutrophil activity [88]. IL-17 responses also drive pathological inflammation following Mtb infection of mice deficient in IFNγ or IL-27 signalling [89–92], and mouse Mtb infection in the context of a chronic viral infection also promotes Th17-mediated neutrophilic immunopathology [93]. In turn, this observation may explain the association between elevated IL-17 activity and hyperinflammatory responses seen with immune reconstitution in HIV [94].

IL-17 induces the expression of traditional neutrophil chemokine molecules (e.g., CXCL8/IL8) as well as calprotectin, a heterodimer of S100A8/9 proteins, and beta defensins, both of which promote neutrophil chemotaxis alongside their antimicrobial properties [95–97]. In Mtb-infected mice, calprotectin promotes neutrophil accumulation and tissue pathology without additional benefit in antimicrobial function [90,98]. S100A8/9 protein expression is observed in human TB granuloma [90], and in the human TST challenge model both S100A8/9 and beta defensins, as well as CXCL8, are overexpressed in response to standardised in vivo antigen stimulation in active TB patients relative to controls [79]. These observations may explain the presence of neutrophils in human TB granuloma [80,99], and accumulation in greater numbers at the site of the human TST challenge in active TB [79]. Human neutrophil stimulation by Mtb can generate neutrophil extracellular traps (NETs) that contain tissue-damaging matrix MMPs [99]. This process, termed NETosis, is closely linked with IL-17, which promotes NETosis and drives further Th17 differentiation, amplifying neutrophil recruitment [100,101]. Importantly, elevated blood neutrophil counts predict radiological severity of lung damage at the end of treatment and mortality in human TB [102,103].

Independent of its role in neutrophil recruitment, IL-17 can also promote tissue damage through the direct induction of MMP secretion by epithelial cells and fibroblasts [104,105]. These enzymes can degrade airway tissue and promote cavitation and are found in elevated levels in the airway of patients with TB disease [106]. The activity of these proteases relates to the extent of human lung disease, and recently use of doxycycline, an inhibitor of MMP activity, as an adjunct to antibiotics showed promise in reducing inflammation and cavitation of pulmonary TB disease [107]. In this context, it is striking that in the human TST challenge model, genes that showed the greatest differential induction in active TB patients compared to controls were MMP-1, -3 and -9, indicating that TB disease may polarise tissue responses to induce further tissue destruction at the site of host-pathogen interactions through the activity of these enzymes [79].

A detrimental role for the IL-17 axis cytokine activity in TB disease is further supported by associations with disease severity. Elevated IL-6 levels and increased frequency of Th17 cells are found in the pleural space of patients with more extensive TB lung lesions [108,109], and stimulation of granuloma-derived T cells induces greater IL-17 production in macaques with extrapulmonary TB dissemination, compared to those with disease restricted to the lungs [110]. IL-17 responses also play a central role in hyper-inflammatory, immune reconstitution responses seen in HIV-TB co-infection [94]. Moreover, the antigen-mediated expansion in blood of IL-17-IFNγ co-expressing Th17.1 cells, strongly associated with tissue pathology in other inflammatory conditions [111–115], correlates with more advanced TB lung disease in humans [116]. In diabetes, which is associated with more radiologically advanced lung disease [117], elevated TB-specific Th17 responses are evident in individuals that fail to control dysglycemia following TB treatment [118], and lack of microbiological clearance at 2 months of TB treatment is specifically associated with elevated circulating IL-17 levels [119].

Although these cross-sectional assessments ultimately cannot truly establish pathological causality, further evidence for excessive inflammation driving TB disease comes from human interventional data. Anakinra, a recombinant form of the human soluble IL-1R, has shown some clinical benefit in dampening excessive inflammation in cases of HIV-associated TB-IRIS [120,121], as well as reduced lung inflammation with no deleterious effect in both TB-infected mice and macaques [122]. Instead, drugs that target the T cell checkpoint molecule PD-1 can unmask and exacerbate TB disease [5,123], and both in vivo and ex vivo attenuation of PD-1 activity is associated with elevated IL-17, as well as IFNγ, responses by CD4+ T cells [116,124–126].

## Model for the role of IL-17 in TB

Collating the polarised arguments presented in this review, we conclude that IL-17 plays a dichotomous role in the context of human Mtb infections. Data from the paucibacillary, subclinical stages of the infection, human genetic polymorphisms, in vitro Mtb infections of myeloid cells, and animal models of early infection or vaccination support IL-17 responses accelerating bacterial clearance and preventing disease. We speculate this occurs by promoting infiltration of activated neutrophils that support bacterial clearance through induction of reactive oxygen species and antimicrobial proteins, although the effect of IL-17 on macrophage microbicidal activity cannot be excluded, possibly in conjunction with other cytokines. Moreover, the precise mechanism by which Th17.1 cells confer protection is not clear, especially whether there is genuine synergy in the activity of IL-17 and IFNγ, or if this phenotype is simply reflective of the functional plasticity of Th17 cell types. Nevertheless, the absence of a TB susceptibility phenotype in individuals with no IL-17 signalling suggests that this cytokine is unlikely to be essential in the resolution of early human Mtb infection or maintenance of latency. In contrast, in the presence of established TB disease, effector functions downstream of persistent and exaggerated IL-17 activity may not provide additional benefit in bacterial clearance but instead promote bystander tissue damage detrimental to host health even beyond antibiotic cure of disease, exemplified by the syndrome of post-TB lung disease (PTLD) [127].

It remains unclear whether IL-17-mediated processes that favour the resolution of early infection are the same as the ones that later drive pathology. This possibility is exemplified by IL-17 and IFNγ co-expressing Th17.1 cells being associated with both granuloma sterilisation in early macaque TB [33,34], and tissue destruction in chronic, non-resolving inflammatory diseases [111–113]. Similarly, in mice, type I IFN, despite promoting neutrophil infiltration, can antagonise macrophage accumulation and CD4 T cell activation, making mice susceptible to TB [128], whereas in established human TB tissue, type I IFN activity may limit the extent of tissue pathology [129]. Both these examples illustrate the importance of interpreting the functional and kinetic effects of cytokines in the context of the wider microenvironment around Mtb infection, which may ultimately determine the balance between protection and pathology.

Overall, we propose that IL-17 activity in TB infections may relate to bacterial burden and granuloma size, with small and/or paucibacillary lesions inducing sufficient IL-17 activity, likely in the context of other cytokines, to enhance additive bactericidal function and contribute to local control. However, rising or persistent bacterial load and granuloma necrosis further amplifies IL-17 responses to the point where benefit from bactericidal activity is greatly outweighed by tissue damage. The resultant chronic inflammatory state may also impact the function of monocytes and their myeloid progenitors [130,131], increasing their potential to promote Th17 differentiation and chemoattract neutrophils [79]. Similar models of disease pathogenesis have been proposed in the granulomatous disease sarcoidosis [132], and may explain the positive association between the size of the Mtb infection inoculum and IL-17 responses in macaques [32].

## Implications for clinical translation

The increased awareness of the role played by IL-17 in TB biology has been harnessed for therapeutic benefit. In vaccination, efforts are now focused on inducing Th17 responses via novel vaccine adjuvants. In particular, the pattern recognition receptor Mincle appears to be crucial for promoting Th17 polarisation [133], and its ligands include trehalose dimycolate (TDM), a major cell wall component of Mtb [134]. A range of synthetic Mincle ligands have been developed that promote Th17 expansion [135], several of which are being explored for TB vaccines. For example, the synthetic Mincle agonist UM-1098 promotes Th17 and Th1 responses in mice when combined with M72 vaccine antigens on silica nanoparticles [136], though protection was inferior to BCG. In addition, Mincle can be combined with other agonists, as in the CAF10b liposome adjuvant, which combines Mincle and a TLR9 agonist. This strongly promotes Th17 and Th1 Mtb-specific response in conjunction with the novel TB vaccine antigen H107 [137] and is currently in a first-in-human trial (ClinicalTrials.gov ID NCT06050356). In addition, several groups have demonstrated that mucosal delivery of various TB vaccines promotes Mtb-specific Th17 responses [29,138,139], which are closely associated with vaccine-induced protection against TB disease [38].

In contrast, the strongest rationale for modulating IL-17 activity in TB disease is to inhibit its function in the context of established disease to minimise immunopathology. In pulmonary TB, the most common disease form, PTLD is increasingly being recognised as a major contributor to chronic lung disease among >155 million TB survivors worldwide, impacting quality of life and economic productivity, but without preventative treatments [127]. Host-directed therapeutic strategies to improve PTLD are currently focussed on interventions that are adjunctive to standard antibiotic therapy [140]. To date no study has proposed direct attenuation of IL-17 activity itself, although monoclonal antibodies that neutralise IL-17A are licenced (secukinumab) and could be repurposed [76]. Indirect attenuation of the IL-17 axis may also prove beneficial, with inhibition of IL-1 or IL-6 activity reducing hyperinflammation and the risk of PTLD by preventing downstream Th17 differentiaon [79,121], whereas direct MMP inhibition in cavitary lung TB diseases may be beneficial by blocking processes downstream of exaggerated IL-17 activity [107]. The benefit of IL-17 blockade may be greatest in extrapulmonary TB where non-specific corticosteroid therapy has known adjunctive therapeutic benefit [141–143]. Ultimately, it’s likely that interventional experimental medicine studies will be required to determine the mechanistic and clinical effects of intervening on the IL-17 axis, as well as the subset of patients that may most benefit [6,144].

## Conclusions

**Fig 1:**
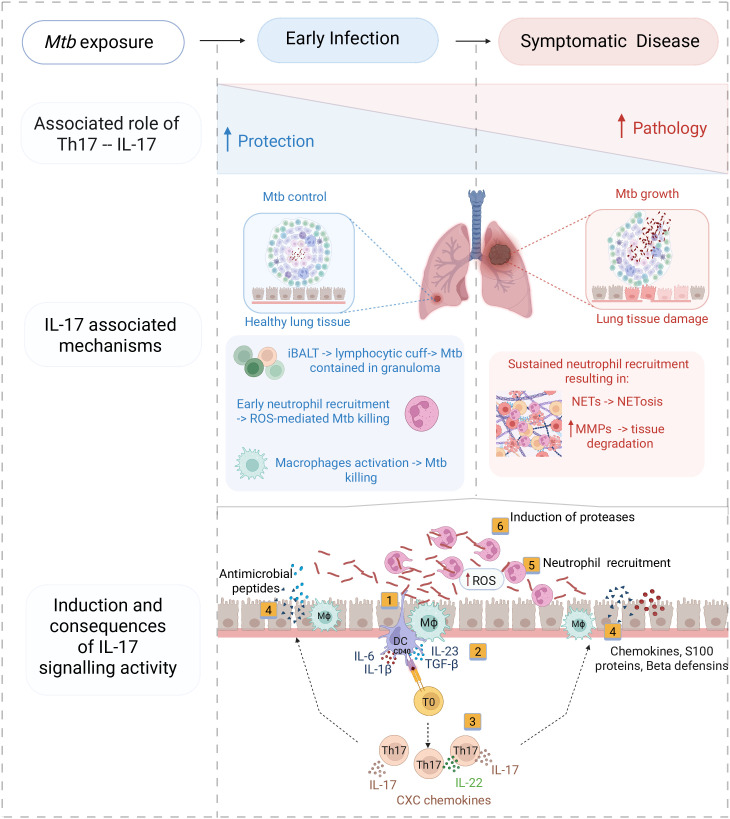
Dual role of IL-17 responses and associated clinical outcomes at different stages of TB. Protective role of Th17 – IL-17 in the early stages of Mtb infection (blue/left) and detrimental effects in the context of symptomatic active disease (red/right), and the IL-17-mediated mechanisms associated with these outcomes. Below, the drivers of IL-17 induction and the functional consequences of the ensuing IL-17 signalling activity: (1) dendritic cells (DCs) and macrophages (Mϕ) immune activation by Mtb, (2) release of IL-23, IL-1β, IL-6 and TGF-β to differentiate Th17 cells from naïve T cells (T0), CD40 expression on DCs is central to enhance Th17 polarisation, (3) release of IL-17 and IL-22 by activated Th17, which (4) triggers release of neutrophil chemokines and antimicrobial peptides from epithelial cells and alveolar macrophages that may control Mtb replication. (5) Neutrophils recruited to the site of infection contribute to ROS production which may be protective, but in established disease may also contribute to (6) induction of matrix metalloproteases that promote tissue damage. Figure generated using BioRender. Maseeme, M. (2026) https://BioRender.com/anzbb6v.

We have summarised the proposed dual role of IL-17 in TB both in Table 1 and Fig 1. Future work will need to define the cellular effectors involved in IL-17 associated protection and pathology, requiring in vivo, ex vivo and in situ assessments at the single-cell level. In addition, it will be important to develop correlates of tissue IL-17 activity to permit their rapid quantification following vaccination, as well as in the context of established TB disease. Nevertheless, our proposed model implies that disease context is key to understanding and harnessing the role of IL-17 in TB. Vaccines that yield IL-17-generating T cell memory responses are likely to contribute to protection from TB disease by promoting rapid eradication of paucibacillary inocula. In contrast, in established TB disease, attenuation of the pathologic functions of IL-17, or cytokines involved in inducing signalling through this axis, such as IL-1 or IL-6, may be beneficial. The use of host-directed biologics that target these cytokines, administered in adjunctive fashion alongside antibiotics, may safely accelerate the resolution of pathological processes, minimise long term tissue damage, and reduce transmission.

**Table 1:** Summary of mechanisms associated with IL-17 activity, and how these impact outcomes in TB.

Mechanisms	Protective	Pathological
Neutrophils	IL-17 induces CXC chemokines and G-CSF to drive early chemotaxis of proinflammatory neutrophils with high bactericidal activity (via ROS production and phagocytosis) limiting Mtb growth. • IL-17 promotes CXC chemokine secretion leading to neutrophil recruitment [97], and ROS induction which supports Mtb killing [12,145]. • Pro-inflammatory neutrophil phenotype aids Mtb control [146].	Sustained IL-17 signalling by extended Mtb exposure in disease leads to chronic CXC chemokine gradients and fibroblast-derived CXCL1 together driving neutrophil-dominant inflammation, excess ROS production and NETosis. • Repeated Mtb exposure increases IL-17 and IL-6 levels, neutrophil infiltration and bacterial burden [88]. • IL-17 triggers lung resident fibroblasts to produce CXCL1, driving persistent neutrophil recruitment and NET formation, which drive lung damage [101]. • Type I IFN-induced NETosis implicated in TB caseating lung damage [147,148].
Mononuclear cells	IL-17 supports bacterial control by PBMC. • IL-17 induced nitric oxide (NO) production in human PBMC, associated with improved bacterial control [ 50].	Sustained IL-17 cytokine and elevated IFNy activity during disease drives macrophage-mediated lung tissue damage. • IL-17 enhances IFNy production, IFNy-driven macrophage polarisation and mucosal pathogenesis [149].
iBALT	IL-17 promotes spatial organisation of immune cells into an early lymphocytic cuff, recruiting Th1 cells and formation of iBALT into a protective granuloma during infection. • IL-17 activity by Th17 cells in the lung promotes inducible bronchus-associated lymphoid tissue (IBALT) [19,23,150].	IL-17 sustained Th1 chemotaxis creates chronic inflammatory microenvironments characterised by excessive proinflammatory cytokines, leading to activation of macrophages and other immune cells. • Lymphoid neogenesis and development of tertiary lymphoid structures cause lung damage in chronic inflammatory lung diseases [151].
Th17 cells	Tissue-resident Th17.1 populations persist at airway sites of pathogen entry, enabling rapid recall response that improves early containment of Mtb. • Th17.1 localised in the lungs associated with Mtb control, and vaccine-induced protection [39,152].	Th17 cell plasticity to Th17.1 differentiation leads to long-term production of IFNy, which impairs Th17 Mtb control observed in chronic disease states. • Co-expression of IFNy by Th17.1 impairs the long-term protective effect of IL-17 cells in TB [152], and promotes tissue pathology in autoimmune disease [113–115].
Secreted factors (antimicrobial peptides & MMPs)	IL-17 induces antibacterial proteins (β-defensin, S100A8/9/calcitonin family) in epithelial cells strengthening the lung mucosal barrier against Mtb. • IL-17 promotes induction of antibacterial proteins such as β-defensin and calprotectin (S100A8/9) in epithelial cells [12,153].	IL-17 drives expression of tissue-degrading proteases in lung tissue resulting in breakdown of extracellular matrix and structural lung damage. • IL-17 linked to induction of tissue degrading MMPs in TB and other diseases [79,104].

**Learning points**

- IL-17 responses prevent disease in early-stage TB infection and following effective vaccination in animal models.
- In established TB disease, persistent and exaggerated IL-17 activity may drive immunopathology.
- Vaccines that generate IL-17-producing T cell memory responses are likely to contribute to protection from TB disease.
- Attenuation of IL-17 activity, or upstream cytokines such as IL-1 or IL-6, may accelerate TB disease resolution and minimise long-term tissue damage.

**Key papers in the field**

1. Mills, K. H. G. IL-17 and IL-17-producing cells in protection versus pathology. Nat. Rev. Immunol. 23, 38–54 (2023).
2. Darrah, P. A. et al. Prevention of tuberculosis in macaques after intravenous BCG immunization. Nature 577, 95–102 (2020).
3. Ogongo, P. et al. Tissue-resident-like CD4+ T cells secreting IL-17 control Mycobacterium tuberculosis in the human lung. The Journal of Clinical Investigation (2021).
4. Cruz, A. et al. Pathological role of interleukin 17 in mice subjected to repeated BCG vaccination after infection with Mycobacterium tuberculosis. J. Exp. Med. 207, 1609–1616 (2010).
5. Pollara, G. et al. Exaggerated IL-17A activity in human in vivo recall responses discriminates active tuberculosis from latent infection and cured disease. Sci. Transl. Med. 13, (2021).
